# Single nucleotide polymorphisms in microRNAs action as biomarkers for breast cancer

**DOI:** 10.3906/biy-2004-78

**Published:** 2020-10-13

**Authors:** Thanh Thi Ngoc NGUYEN, Minh Thi Hong TRAN, Vy Thi Lan NGUYEN, Uyen Doan Phuong NGUYEN, Giang Dien Thanh NGUYEN, Luan Huu HUYNH, Hue Thi NGUYEN

**Affiliations:** 1 Department of Physiology and Animal Biotechnology, Faculty of Biology and Biotechnology, University of Science, Ho Chi Minh City Vietnam; 2 Vietnam National University, Ho Chi Minh City Vietnam

**Keywords:** Breast cancer, microRNA, miRNA, single nucleotide polymorphism, SNP

## Abstract

MicroRNAs (miRNAs) have been recently described as small noncoding RNAs that are involved in numerous crucial physiological processes, such as cell cycles, differentiation, development, and metabolism. Thus, dysregulation of these molecules could lead to several severe disorders, including breast cancer (BC). Ongoing investigations in malignant growth diagnostics have distinguished miRNAs as promising disease biomarkers. As with any other mRNAs, single nucleotide polymorphisms (SNPs) in DNA sequence encoding for miRNA (miR-SNPs) indeed lead to potential changes in the function of miRNA. In this study, miR-SNPs located in different miRNA sequence regions, which have been associated with BC in different ways, and the potential mechanisms of how these miR-SNPs develop the risk of the disease were discussed.

## 1. Introduction

Breast cancer (BC) is the most widely recognized disease among women worldwide. Together with several identified penetrance BC predisposition genes, microRNAs (miRNAs) have gained more attention due to their relationship with BC development (Wightman et al., 1993). MiRNAs are negative regulators of gene expression acting on mRNA (Catalanotto et al., 2016). They have been implicated in various physiological processes, such as development, metabolism, differentiation, proliferation, and responses to stress (Hu and Coller, 2012; Iwakawa and Tomari, 2015). The bioinformatics data system of about 2500 known mature miRNAs in human was published, with an estimation of more than 60% mRNAs in the mammalian genome being regulated by miRNAs (Stoicea et al., 2016).

MiRNAs are single-stranded noncoding RNA molecules of approximately 22–24 nucleotides in length that regulate posttranscriptional gene expression (Starega-Roslan et al., 2011). The biogenesis of miRNAs (O’Brien et al., 2018) starts in the nucleus, where primary miRNA (pri-miRNA) is first transcribed from its gene (miRNA gene), is cleaved, and then modified to form precursor miRNA (pre-miRNA). This precursor is then transported to the cytoplasm to join in another process that generatesan imperfect miRNA duplex that contains the mature miRNA strand andits complementary strand, miRNA*. Through a separation process, the miRNA* strand is degraded and only a mature miRNA strand remains. Finally, the mature miRNA comes into contact with the argonaute protein of the RNA-induced silencing complex (RISC) to form a stable active miRNA-RISC complex that can specifically bind to target mRNA and promote the translational inhibition or degradation of this mRNA.

Single nucleotide polymorphisms (SNPs) occur as frequently as 1 per 1 kbp in the genome (Shastry, 2009). Among these SNPs, those that lie within miRNA genes (miR-SNPs) have been found to be involved in the interpretation of the essential transcript, the processing of pri-miRNA and pre-miRNA and the interactions between miRNA and mRNA. MiR-SNPs in seed regions of miRNAs, which are regions liable for the binding between miRNA and mRNA, can interrupt miRNA binding or be destructive. Moreover, SNPs can make new connections between miRNA seed regions and target mRNA by changing the sequence homology (Chen et al., 2008). Polyadenylation, protein-mRNA connections, and miRNA-mRNA interactions can be modified by miR-SNPs in the untranslated regions (3′UTRs) of mRNA. These alterations can effectively affect mRNA stability and translation efficiency (Nicoloso et al., 2010). Correspondingly, SNPs in primary and precursfor miRNA sequences can influence miRNA production and stability, as well as miRNA-mRNA interactions, prompting changes in the expression of miRNA target genes (Ryan et al., 2010). Despite missing biological validation, case-control studies have provided proof of the relationship of miR-SNPs and the risk of cancer. These investigations have contrasted at the level of practical support regarding the expected interaction, mechanical understanding, and validation status.

Such miR-SNPs can cause an increased risk of several cancers, including BC (Zheng et al., 2011). All sorts of miR-SNP can be potentially novel biomarker candidates for disease susceptibility testing, personalized prognosis, and the clinical treatment of BC patients (Hoffman et al., 2009; Sung et al., 2012; Ryan, 2017). In this study, the critical role of miR-SNPs and their potential mechanisms in BC will be discussed.

## 2. SNPs in pri-miRNA sequence and BC

The hairpin double-stranded stem structure, along with a terminal loop and the 2 single-stranded flanking regions of pre-miRNAs play a crucial role in the processing executed by the Drosha-DCGR8 complex (Wahid et al., 2010). Therefore, SNPs that are located within these regions of the miRNA genes may alter molecular processing and affect miRNA maturation (Han et al., 2004; Zeng and Cullen, 2005; Sun et al., 2009; Auyeung et al., 2013; Cammaerts et al., 2015). In addition, transcriptional elements like the promoter regions have been reported to be present in pri-miRNA sequences (Song Gao et al., 2010). Hence, the presence of any variant within the long flanking sequence of pri-miRNA suggests an alteration in the recruitment of necessary factors required for miRNA transcription. Thus far, it has been suggested that functional SNPs in pri-miRNA sequences (pri-miR-SNPs) are related to the processing of mature miRNA and risk of cancer. In a study of SNPs in primary transcripts of miRNAs, 4 SNPs in pri-miR-3065 showed the possibility of being associated with BC. Particularly, the impairedmiR-3065 processing caused by these 4 SNPs lead to the downregulation of mature miR-3065 and reduced the inhibition of its target oncogenes (Bensen et al., 2018).

In this study, 4 other SNPs, rs353291, rs1053872, rs4284505, and rs12976445, in the primary transcripts of 4 different miRNAs were discussed in terms of their effects on mature miRNA levels, the expression of their target genes, and their relationship with the risk of BC (Table).

**Table:** Effect of the risk allele of miR-SNP on the BC risk via regulation of the expression of miRNA and related genes

SNP-risk allele	SNP position	Affected miRNA expression	Related gene expression	BC risk association study	Reference
rs353291-G	Pri-miRNA sequence	↓ miR-145	↑ EGFR	Increased risk	Cho et al. (2011), Yan et al. (2014), Chacon-Cortes et al.(2015a), Duan et al. (2020)
rs1053872-G		↓ miR-101	↑ STMN1, ↑ CXCR, ↑ JACK2	Increased risk	Wang et al. (2012), Chen et al. (2014a), Wang et al. (2014)
rs4284505-G		↓ miR-17HG	↑ CCDN1	Increased risk	Yu et al. (2008), Chacon-Cortes et al.(2015b)
rs12976445-C		↓ miR-125a	↑ ERBB2	Increased risk	Schulman et al. (2005), Wu and Belasco (2005), Scott et al. (2007), Lehmann et al. (2013), Jiao et al. (2014), Yamagata et al. (2014)
rs895819-C	Pre-miRNA sequence	↓ miR-27a	↑ FBW7	Decreased risk	Zeng et al. (2005), Yang et al. (2010), Lerner et al. (2011), Xiong et al. (2013), Zhang et al. (2013), Dai et al. (2015), Zhang et al. (2017b)
rs2682818-A		↓ miR-618	↓ PTEN	Increased risk	Fu et al. (2014), Morales et al. (2016), Feng et al. (2019)
rs6505162-C		↓ miR-423	↑ CDKN1A	Increased risk	Lin et al. (2011), Smith et al. (2012), Zhao et al. (2015), Morales et al. (2016), Wazir et al. (2019)
rs2043556-G		↓ miR-605	↑ MDM2	Increased risk	Xiao et al. (2011), Chen et al. (2014b), Zhou et al. (2014), Said and Malkin (2015), Morales et al. (2018), Kazemi and Vallian (2020)
rs4919510-G	Mature miRNA sequence	↓ miR-608	↑ HSF1	Increased risk	Cheng et al. (2012), Huang et al. (2012), Hashemi et al. (2016)
rs11614913-C		↑ miR-196a2	↓ HOXD10, ↑ TOX3	Increased risk	Easton et al. (2007), Ma et al. (2007), Stacey et al. (2007), Hoffman et al. (2009), Hu et al. (2009), Qiu et al. (2011), Minh et al. (2018)
rs3746444-G		↓ miR-499a	↑ SOX	Increased risk	Alshatwi et al. (2012), Li et al. (2013), Yan et al. (2017), Zhang et al. (2017a)
rs2910164-C		↑ miR-146a	↑ TRAF6	Increased risk	Deng (2006), Shen et al. (2008), Lian et al. (2012), Dai et al. (2015), Minh et al. (2018)

↑ upregulation; ↓ downregulation

### 2.1. Effect of rs353291 (A/G) on pri-miR-145 in breast tumorigenesis

SNP rs353291 is located in pri-miR-145, 450 bp upstream of the miR-145 gene. One of thesemiR-145 targets is epidermal growth factor receptors (EGFRs) (Cho et al., 2011), which are involved in cell proliferation and have been well-proven to be related to BC (Yan et al., 2014). There have been 2 possible mechanisms found through which rs353291 can be involved in BC. First, the SNP lies within the binding site of transcriptional factors. Thus, the variation of rs353291 may decrease miR-145 expression, leading to an increase in the expression level of its oncogenic target gene, and finally resulting in tumorigenesis. In fact, a study found that the expression level of miR-145 was significantly downregulated in a variety of tumors (Cho et al., 2011). Moreover, the presence of the G allele at rs353291 was shown to increase the risk of developing BC in 2 independent case-control Australian Caucasian populations [G vs. A: odd ratio (OR) = 1.37, 95% confidence interval (95% CI) = 1.01–1.84, P = 0.041 and OR = 1.31, 95% CI = 1.04–1.70, P = 0.023] (Chacon-Cortes et al., 2015a). Hence, the G variant of rs353291 might have a role in decreasing miR-145 expression. In the second mechanism, rs353291 might indirectly cause abnormalities in the length of telomeres. GG-genotype individuals were found to have longer telomere when compared with those who had AA or AG genotypes (P = 0.005) (Duan et al., 2020). Both shorter and longer telomere can lead to cancers (Aviv et al., 2017; McNally et al., 2019). Therefore, it is possible that the G variation of rs353291 may first decrease miR-145 expression which, in turn, increases the expression level of longer-telomere association gene, resulting in the increased risk of BC.

### 2.2. Effect of rs1053872 (C/G) on pri-miR-101 in breast tumorigenesis 

SNP rs1053872 is located in pri-miR-101-2, 10 kb downstream from the miR-101-2 gene. This SNP has been predicted to be in the extended sequence flanking 3′ side of the pre-miR-101, which may affect the cleavage of Drosha and correspondingly, the processing of mature miR-101-3p. In fact, the G allele carriers were shown to be significantly associated with the increased risk of BC [(CG + GG vs. CC: OR = 1.179, 95% CI = 1.040–1.337, P = 0.010) (Chen et al., 2014a)] and miR-101-3p have been revealed to be downregulated in BC (Wang et al., 2012; 2014). Therefore, the G allele of rs1053872 might be related to the inhibition of miR-101-3p maturation in BC. Although the precise mechanism of miR-101-3p in BC regulation is not entirely clear, miR-101-3p was observed to inhibit proliferation, invasion, and metastasis via targeting Stathmin1 (STMN1) and C-X-C chemokine receptor type 7 (CXCR7)(Wang et al., 2012; Li et al., 2015) and promote apoptosis by targeting Janus kinase 2 (JAK2) in BC cells (Wang et al., 2014). 

### 2.3. Effect of rs4284505 (A/G) on pri-miR-17HG in breast tumorigenesis 

SNP rs4284505 is located in the intron of the miR-17 host gene (miR-17HG), 2kb upstream of miR-17 and miR-18a genes. Intronic variants have been demonstrated to affect the transcription, the splicing proficiency of their host genes, and the expression of different transcripts. Additionally, some variants may influence the expression of distant genes. Therefore, rs4284505 might affect the processing of miR-17HG, which has been known as an essential regulator of genes involved in BC pathways, such as upregulating (Cyclin D1) CCND1 (Yu et al., 2008). A study from Chacon-Cortes et al. revealed that a rs4284505 variant of miR-17HG was closely related to the risk of BC in 2 Australian case-control populations (P = 0.01 and P = 0.03), and the presence of the A allele appeared to have a protective effect on susceptibility to BC (Chacon-Cortes et al., 2015b). Therefore, the A allele of rs4284505 might increase the miR-17HG expression.

### 2.4. Effect of rs12976445 (C/T) on pri-miR-125a in breast tumorigenesis 

SNP rs12976445, which was found to be significantly associated with worse survival in BC (P = 0.04) (Jiao et al., 2014), is located in pri-miR-125a, 54bp upstream of the miR-125a gene. How rs12976445 variants affect the processing of pre-miR-125a remains to be elucidated. One hypothesis was that rs12976445 may interfere with the interaction between Drosha and pri-miRNA, subsequently reducing miR-125a maturation or affecting miR-125a level (Yamagata et al., 2014). Studies have shown that the SNP was linked to a decline in miR-125a levels. For instance, Lehmann et al. found that the samples that had allele C of SNP rs12976445 (either heterozygous or homozygous) exhibited a decrease of 85% (P = 0.024) in the miR-125a level when compared with those that contained TT variant (Lehmann et al., 2013). Another study conducted by Scott et al. revealed that miR-125a was significantly downregulated in *Erb-B2 receptor tyrosine kinase 2* ( *ERBB2* )-amplified BCs (Scott et al., 2007). Notably, tumor samples with the CC or CT genotype of rs12976445 seemed to have had higher *ERBB2* mRNA level than those with the TT genotype, with an increase of 2.4-fold (P = 0.044) (Lehmann et al., 2013). Because *ERBB2* is one of the target genes of miR-125a (Schulman et al., 2005; Wu and Belasco, 2005; Scott et al., 2007), it might be possible that the CC or CT genotype of rs12976445 can increase the risk of BC via the downregulation of miR-125a,followed by the upregulation of *ERBB2* . 

## 3. SNPs in pre-miRNA sequence and BC

In the pre-miRNA structure, the sequence at 2-nucleotide overhanging the unpaired flanking arms and within the hairpin are essential for the accurate binding of Exportin-5/Ran-GTP complex and Dicer (Yi et al., 2003; Kim, 2004; Zhang et al., 2004; Tsutsumi et al., 2011; Gu et al., 2012; Tian et al., 2014). Therefore, SNPs located within pre-miRNA sequences (pre-miR-SNPs) could lead to a shift in the processing sites during the miRNA/miRNA* duplex biogenesis, resulting in the alteration of mature miRNAs and subsequently, target mRNAs.

In BC, 44 pre-miR-SNPs (rs2682818, rs6505162, rs2043556, and rs895819) were found to have an effect on the level of their mature miRNAs. The alterations of miRNA expression consequently upregulated the expression of oncogenic target genes or downregulated the expression of tumor suppressor genes, resulting in as increased risk of BC. The potential mechanisms of pre-miR-SNPs associated with the risk of BC affecting the mature miRNAs and target gene expressions are summarized in the Table.

### 3.1. Effect of rs895819 (A/C) on pre-miR-27a in breast tumorigenesis 

**Figure 1:**
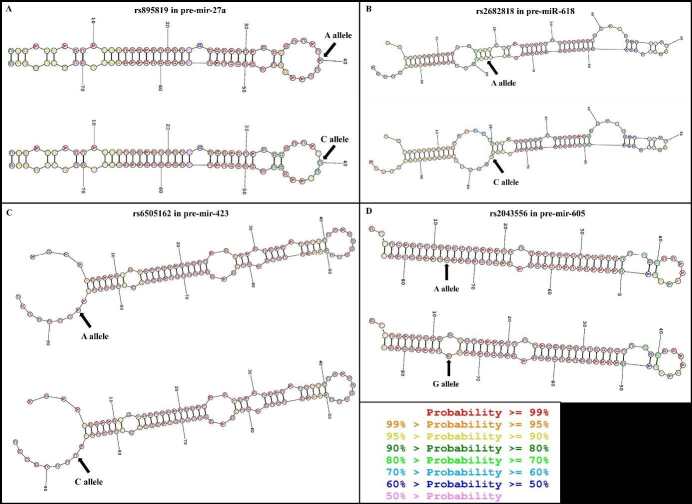
Predicted secondary structure of pre-miRNA with 2 alleles of pre-miR-SNP. Sites of the SNPs are indicated with arrows. This figure was predicted using the webserver at https://rna.urmc.rochester.edu/RNAstructureWeb/.

SNP rs895819 (A/C) is in pre-miR27a and is located in the terminal loop (Figure 1). Hence, this SNP can change the size of the terminal loop and affect the binding of some components of the Drosha or Dicer complexes, such as KSRP, thus interfering with this miRNA maturation (Zeng et al., 2005; Yang et al., 2010). In fact, studies by Zeng et al. and Xiong et al. showed that the C allele of rs895819 caused a decrease in pre-miR-27a products, as well as mature miR-27a expression (Zeng et al., 2005; Xiong et al., 2013), leading to an upregulation of the F-box/WD repeat-containing protein 7 (FBW7) (Lerner et al., 2011; Xiong et al., 2013), which in turn downregulated multiple oncoproteins and reduced the chance of tumorigenesis (Lerner et al., 2011).

The protective impact of the C allele on BC development has been confirmed in several case-control studies, including in a Chinese population [C vs. A: OR = 0.628, 95% CI = 0.410–0.961, (Zhang et al., 2013); C vs. A: OR = 0.93, 95% CI = 0.87–0.99 (Zhang et al., 2017b)], German population [C vs. A: OR = 0.88, 95% CI = 0.78–0.99, P = 0.0287 (Yang et al., 2010)], and in all of the populations in a meta-analysis [C vs. A: OR = 0.91, 95% CI = 0.85–0.98, P = 0.02 (Dai et al., 2015)]. SNP rs895819 was also associated with the risk of BC in BRCA2 mutation carriers (Kontorovich et al., 2010), which indicated that this SNP may be involved in familial BC development.

### 3.2. Effect of rs2682818 (A/C) on pre-miR-618 in breast tumorigenesis 

SNP rs2682818 (A/C) is located in pre-miR-618. The A allele has been predicted to change the first internal stem-loop of miR-618 and its structure contains a small internal loop at the Drosha processing site (Figure 1). This SNP position may affect Dicer and Drosha processing, which may alter the production of pre-miR-618 and mature miR-618. Indeed, Fu et al. (2014) reported that the Aallele was associated with the decreased level of mature miR-618. The decrease in mature miR-618 levels may initiate BC tumorigenesis by deregulating miR-618-dependent pathways. Phosphatase and tensin homolog (PTEN), a BC-associated protein, was one of the predicted targets of miR-618. The A allele reduced the binding affinity of miR-618 to the 3′UTRs of PTEN and thus, might deactivate the tumorigenesis. The enhanced risk of BC of miR-618 rs2682818 variant was revealed. The A allele carriers were observed to be associated with increased risk of BC [A vs. C: OR = 1.3, 95% CI = 1.0−1.8, P = 0.03; CA + AA vs. CC: OR = 1.4, 95% CI = 1.0−2.0, P = 0.02 (Morales et al., 2016)] in a South American population. In a meta-analysis, an increased risk of BC caused by rs2682818 was discovered in heterozygote genetic model (AC vs. CC: OR = 1.291, 95% CI = 1.012–1.648, P = 0.040) and dominant contrast model (AA + AC vs. CC: OR = 1.280, 95% CI = 1.009–1.623, P = 0.042) (Feng et al., 2019).

### 3.3. Effect of rs6505162 (A/C) on pre-miR-423 in breast tumorigenesis 

SNP rs6505162 (A/C) resides within the single-stranded flanking segments of pre-miR-423, 12 bp from the cleavage site of DGCR8 and approximately 11 bp from the stem-ssRNA junction (Han et al., 2006). Although the A and the C alleles of rs6505162 have been predicted to have the same structure (Figure 1), the SNP was hypothesized to alter the maturation of miR-423 due to its proximate location from the DGCR8 cleavage site. In fact, the C allele of rs6505162 was found to be involved in a process that prevents pri-miR-423 from being normally processed into 2 mature miRNAs (Zhao et al., 2015). In addition, the mature miR-423 was reported to downregulate cyclin-dependent kinase inhibitor 1A (CDKN1A) (p21Cip1/Waf1) expression by directly targeting the 3′UTR of CDKN1A (Lin et al., 2011), which functions as a key inhibitor of S-phase DNA synthesis and cell cycle progression. A significantly direct correlation was found in cancerous tissue between telomerase reverse transcriptase and CDKN1Ain the context of human BC (Wazir et al., 2019). Therefore, rs6505162 might be related to the development of BC via miR-423 and CDKN1A.

Indeed, the CC genotype of rs6505162was observed to reduce the risk of BC development (CC vs. AA: OR = 0.50, 95% CI = 0.27–0.92, P = 0.03) in Caucasian Australian women (Smith et al., 2012). Furthermore, the AC genotype of rs6505162 was shown to be related to increased risk of BC in BRCA2 carriers (Kontorovich et al., 2010), which indicated that this SNP may have a role in familial BC. A study of South American women also found that the A allele and AA genotype of rs6505162 were significantly associated with increased risk of familial BC in people with a strong family history of BC (A vs. C: OR =1.3, 95% CI = 1.0–1.7, P = 0.04; AA vs. CC: OR = 1.7, 95% CI = 1.0–2.0, P = 0.05) (Morales et al., 2016). Interestingly, a study in Saudi Arabia found that the third allele of this SNP (allele T) was associated with increased BC susceptibility (T vs. C: OR = 2.63, 95% CI = 1.77–3.91, P = 0.001) (Mir et al., 2018). These findings suggested that the non-C allele of rs6505162 may increase the level of mature miR-423, thereby inhibiting the expression of CDKN1A and inducing breast tumorigenesis.

### 3.4. Effect of rs2043556 (A/G) on pre-miR-605 in breast tumorigenesis 

SNP rs2043556 (A/G) is located in the precursor region of the miR-605 gene and influences the processing of the miRNA. The G allele of rs2043556 has been predicted to create an additional internal loop around the cleavage site of Dicer, while the A allele created a complementary loop (Figure 1). The structural change caused by the G allele implies a possible role of it in decreasing mature miR-605 levels (Said and Malkin, 2015). Furthermore, miR-605 has been predicted to inhibit major oncogene expression (Kazemi and Vallian, 2020), including the validated one, *murine double minute 2* ( *MDM2* ) (Xiao et al., 2011; Zhou et al., 2014). In light of the possibility that the G allele of rs2043556 may promote BC progression via the upregulation of relevant oncogenes, studies have been conducted to examine whether miR-605 rs2043556 was associated with the risk of BC. The G allele was proven to significantly increase the risk of BC in Iranian [G vs. A: OR = 2.159, 95% CI = 1.56–2.99, P = 3 Í 10-6 (Kazemi and Vallian, 2020)], Asian (Chen et al., 2014b), and South American (Morales et al., 2018) populations.

## 4. SNPs in mature miRNA sequence and BC

The miRNA-RISC complex is guided to the complementary sequences in the 3′UTR of target transcripts by mature miRNAs. The site-specific mRNA is cleavaged when the pairing is almost complete, or the translation is inhibited when imperfect base pairing occurs (Sun et al., 2009). Base pairing between 6 or 7 consecutive nucleotides in the target mRNAs 3′UTR and when the seed sequence of the miRNAs 5′ end (2–7 or 2–8 nucleotides) play a critical role in the interactions of miRNA and mRNA. The available findings suggested that any variant in the seed sequence could change the level of their miRNA and might also generate a novel miRNA, which would then affect their downstream targets.

In BC, 4 SNPs in the mature miRNA sequence (miR-SNPs), rs4919510, rs11614913, rs3746444, and rs2910164, were found to be associated with the risk of the disease. The potential effects of these miR-SNPs on the miRNA level and the expression of their target genes are shown in the Table.

In general, all of these SNPs might affect not only their target genes, but also their mature miRNA levels, and therefore potentially induce the increased risk of BC.

### 4.1. Effect of rs4919510 (C/G) on mature miR-608 in breast tumorigenesis 

SNP rs4919510 (C/G) is located in the mature miRNA region of miR-608. The G allele of the SNP has been shown to be associated with the risk of BC in Iranian [G vs. C: OR = 0.53, 95% CI = 0.30–0.92, P = 0.024 (Hashemi et al., 2016)] and Chinese [G vs. C: OR = 1.62, 95% CI = 1.23−2.15, P = 3.4 × 10–4 (Huang et al., 2012)] populations. 

**Figure 2:**
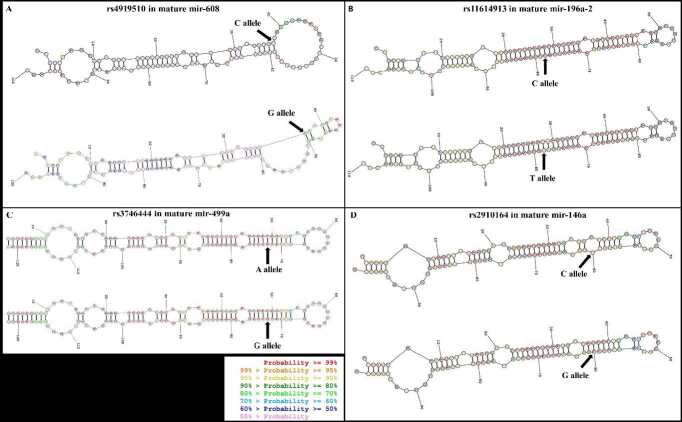
Predicted secondary structure of pre-miRNA with 2 alleles of miR-SNP in the mature region. Sites of the SNPs are indicated with arrows. This figure was predicted using the webserver at https://rna.urmc.rochester.edu/RNAstructureWeb/.

This association can be explained by the effect on their target gene, *heat shock transcription factor-1* ( *HSF1* ). The G allele of rs4919510 was found to have a lower affinity to the binding sites of HSF1 3′UTR (Huang et al., 2012), indicating an increase in the level of HSF1. This high level of HSF1 was found to be related to the risk of BC (Cheng et al., 2012). Moreover, in the G-allele predicted structure (Figure 2), the terminal loop had completely changed, which might have affected the recognition and cleavage of Dicer, which may alter the mature products of miR-608 and in turn, affect the level of HSF1.

### 4.2. Effect of rs11614913 (C/T) on mature miR-196a2 in breast tumorigenesis 

SNP rs11614913 (C/T) is located in the 3p-strand mature sequence of miR-196a2. The increased risk of BC of the C allele of rs11614913 was reported in Chinese [CC vs. TT: OR = 1.37, 95% CI = 1.08–1.74, P = 0.011 (Hu et al., 2009)], Vietnamese [C vs. T: OR = 1.28, 95% CI = 1.02–1.61, P = 0.033; CC vs. CT + TT: OR = 1.64, 95% CI = 1.15–2.35, P = 0.006 (Minh et al., 2018)], Asian, and Caucasian [CC vs. TT: OR = 1.30, 95% CI = 1.01–1.68, P = 0.028 (Qiu et al., 2011)] populations.

The miRNA structure prediction (Figure 2) found a minor change, wherein 2 bases in the variant position in the C allele structure (≥ 99%) were more probable than in the T allele structure (90%–94%), indicating the higher possibility of an extra internal loop formation in the T allele structure. The presence of an additional loop in this position might suppress the processing by Dicer into the mature form of miR-196a2. In a study where a comparison was made between 2 variants of the SNP rs11614913, BC cells transfected with the C allele exhibited a higher mature miR-196a2 level than those transfected with the T allele (Hoffman et al., 2009). The increased level of miR-196a2 with the C allele might inhibit the expression of *homeobox D10* ( *HOXD10* ) and enhance TOX high mobility group box family member 3 (TOX3)/trinucleotide repeat-containing 9 (TNRC9) expression, which have roles in the invasion, metastasis, and DNA repair progress in BC (Easton et al., 2007; Ma et al., 2007; Stacey et al., 2007).

### 4.3. Effect of rs3746444 (A/G) on mature miR-499a in breast tumorigenesis 

SNP rs3746444 (A/G) is located at the 3p and 5p mature miRNA regions of miR-499a. In the RNA structure prediction, there was no difference in the secondary structure (Figure 2). However, the G allele of this SNP was proven to be significantly associated with increased BC susceptibility in Asian and Caucasian populations [G vs. A: OR = 1.17, P = 0.008 (Zhang et al., 2017a); G vs. A: OR = 1.18, 95% CI = 1.09–1.27, P = 0.04 (Yan et al., 2017)]. Furthermore, a decreased miR-499a-5p expression was observed in breast tissues with the G allele of rs3746444 (Alshatwi et al., 2012). The hypothesis was that the G allele of rs3746444 might decrease the miR-499a-5p expression (under an unknown mechanism), which may increase Sry-type HMG box (SOX) expression (Li et al., 2013) leading to the activation of the Wnt/β-catenin signaling pathway in an abnormal manner, which has been associated with breast tumorigenesis and tumor progression.

### 4.4. Effect of rs2910164 (G/C) on mature miR-146a in breast tumorigenesis 

SNP rs2910164 (G/C) is located in the internal loop and mature region of the miR-146a gene. Previous findings have indicated that the C allele of the SNP rs2910164 increased the risk of BC among European [OR = 1.29, 95% CI = 1.02–1.63, P = 0.032 (Lian et al., 2012)] and Caucasian [OR = 1.31, 95% CI = 1.05–1.65, P = 0.02 (Dai et al., 2015)] populations. In 2018, a study conducted in a Vietnamese population also revealed an increased risk among CG genotype carriers [CG vs. CC + GG: OR = 1.44, 95% CI = 1.04–1.98, P = 0.027 (Minh et al., 2018)]. However, the heterozygous variant GC and the C allele were associated with reduced risk in a North Indian population [GC vs. GG: OR = 0.5, 95% CI = 0.31–0.87, P = 0.013; C vs. G: OR = 0.6, 95% CI = 0.4–0.8, P = 0.01 (Bansal et al., 2014)]. The evidence from the published literature clearly showed that rs2910164 in pre-miR-146a may contribute to genetic susceptibility to BC. However, the relationship varied among different populations, which may have been due to interaction with other genetic factors in each population.

By predicting the impact of the variant (C/G) of rs2910164 on the secondary structure of the miR-146a precursor, the C allele was found to create 2 internal loops around the cleavage site of Dicer, while the G allele created only 1 internal loop (Figure 2). This difference implied that the C allele might generate more mature miR-146a than the G allele, which was confirmed in the study of Shen et al. (2008) (P = 0.013). Due to the high level of miR-146a, the C allele structure could inhibit more BC 1 (BRCA1) mRNA than the G allele (P = 0.046) (Shen et al., 2008). Downregulation of BRCA1 reduces the DNA repair function and allows damage cells to progress into S phase, resulting in breast tumorigenesis (Deng, 2006). 

## 5. Conclusion

MiRNAs are small RNAs that regulate various fundamental cell processes by suppressing the expression of their target genes. All types of miR-SNPs can contribute to the development of the malignant tumors, including BC. Case-control studies in different populations have been able to distinguish specific miR-SNPs and predict their effects on the risk of BC for each ethnicity. Therefore, miR-SNPs may act as predictive markers for the risk of BC to support the improvement of early diagnosis and prognosis strategies.

**Acknowledgments**

This research was funded by Vietnam National University, Ho Chi Minh City (VNU-HCM) under grant number 562-2020-18-02. The authors would like to thank the Oncology Hospital HCMC for their contribution to collecting the samples.
